# Longitudinal Study of Sensory Features in Children with Autism Spectrum Disorder

**DOI:** 10.1155/2017/1934701

**Published:** 2017-08-27

**Authors:** Lucia Perez Repetto, Emmanuelle Jasmin, Eric Fombonne, Erika Gisel, Mélanie Couture

**Affiliations:** ^1^École de Réadaptation, Faculté de Médecine et des Sciences de la Santé, Université de Sherbrooke, Sherbrooke, QC, Canada J1H 5N4; ^2^Axe Mère-Enfant, Centre de Recherche du Centre Hospitalier Universitaire de Sherbrooke (CRCHUS), Sherbrooke, QC, Canada J1H 5N4; ^3^Axe le Développement de l'Enfant dans sa Famille et sa Communauté, Institut de Première Ligne en Santé et Services Sociaux, CIUSSS de l'Estrie-CHUS, Sherbrooke, QC, Canada J1H 4C4; ^4^Department of Psychiatry and Institute for Development and Disability, Oregon Health & Science University, Portland, OR 97239, USA; ^5^Center for Interdisciplinary Research in Rehabilitation, Montreal, QC, Canada H2H 2N8; ^6^School of Physical & Occupational Therapy, Faculty of Medicine, McGill University, Montreal, QC, Canada H3G 1Y5

## Abstract

**Background:**

Between 45 and 95% of children with Autism Spectrum Disorder (ASD) present sensory features that affect their daily functioning. However, the data in the scientific literature are not conclusive regarding the evolution of sensory features in children with ASD. The main objective of this study was to analyze the sensory features of children within the age of 3-4 (T1) when they received their ASD diagnosis and two years later (T2) when they started school.

**Methods:**

We conducted a prospective cohort study to assess sensory features in 34 children with ASD over time. The data were collected using a standardized assessment tool, the Sensory Profile.

**Results:**

Our analyses show that sensory features in children with ASD are stable from the age of three to six years. The stability of sensory scores is independent of correction by covariates, such as cognitive level and autism severity scores.

**Conclusions:**

Children with ASD have sensory features that persist from the time of diagnosis at the age of 3 to 4 years to school age. This persistence of sensory features from an early age underscores the need to support these children and their parents. Sensory features should be detected early and managed to improve functional and psychosocial outcomes.

## 1. Introduction

Autism Spectrum Disorder (ASD) affects more than 1% of children [1]. Common characteristics associated with ASD are difficulties in communication and social interaction, as well as rigidity or repetitiveness in interests and behaviors [2]. Some restricted or repetitive interests and behaviors of people with ASD may be related to sensory features [3], such as difficulties in sensory processing and sensory integration [4–6].

Sensory processing is the process by which peripheral and central nervous systems receive, interpret, and respond to sensory information [7, 8]. The nature of the sensory input can vary (smell, touch, proprioception, etc.) and the organization of this information contributes to the development of muscle tone, motor skills, self-awareness, interactions with others, and everyday functioning [9–11]. According to Dunn's model of sensory processing [12], four sensory processing patterns characterize the perceptual process. These patterns are thought to arise from individual differences in neurological thresholds for stimulation (high-low) and self-regulation strategies (active-passive). Crossing these dimensions gives four sensory processing styles: sensory sensitivity, sensory avoiding, low registration, and sensory seeking [12, 13].

With advances in research, difficulties in sensory processing in children with ASD are becoming increasingly evident. Between 45 and 95% of children with ASD have sensory features that affect their functioning in everyday life [14, 15]. However, the data available in the scientific literature shed little light on the evolution of sensory features in children with ASD, making it impossible to provide appropriate services adapted to their specific needs. In addition, few longitudinal studies have been conducted on the sensory development of children with ASD [16, 17] and none studied ASD children prospectively for more than one year under stable conditions, that is, with every child in the study having the same age at the beginning of the study, as well as using the same assessment instruments.

To illustrate the aforementioned lack of conclusive evidence, we briefly reviewed the studies that documented the evolution of overall sensory features (including all senses) of children with ASD. First, a recent prospective study by McCormick et al. (2016) [17] found that 29 children aged 2 to 8 with ASD showed no significant change in sensory processing over time. The main limitations of this study are as follows: (1) only the Short Sensory Profile (SSP) [18] was used, limiting the interpretation of the subcategories (sections and quadrants) and not allowing interpretation according to the quadrants in Dunn's model of sensory processing [12]; and (2) the time between the two evaluations varied, which added a maturation bias.

A second longitudinal study by Ausderau et al. (2014) [16] analyzed the sensory features of 884 children with ASD aged 2 to 12 over one year. This study found no statistically significant differences in the sensory features of these children over time. However, no subdivisions in the wide age range (10 years) of the study participants were analyzed, which limits the interpretation of the results.

A third longitudinal study by Green et al. (2012) [19] included 149 participants with ASD from 18 to 30 months old at the beginning of the study and reevaluated them one year later. The evaluation tool used for this study, the Infant Toddler Social Emotional Assessment (ITSEA) [20], has a sensory component but was not specifically designed to assess sensory features. The ITSEA mainly examines emotional responses, sensory overresponsiveness, and anxiety. Sensory overresponsiveness in the study sample was found to be stable over time.

The findings of these three papers are in contradiction with those of the meta-analysis by Ben-Sasson et al. [21], which analyzed 14 cross-sectional studies involving children with ASD at different ages. This review revealed an increase in the frequency of overall sensory features up to 6–9 years of age (and a decrease thereafter) in children with ASD.

The weaknesses of this meta-analysis are as follows: (1) the inclusion of studies with heterogeneous groups of participants with age ranging from 7 months to 56 years; (2) use of a variety of evaluation tools; (3) diagnoses not all made alike as only 4 of the 14 studies reviewed used gold standard diagnostic tools for ASD; and (4) few studies reviewed including covariates in the analyses.

Other longitudinal studies exist. However, some focused on a single sensory system (e.g., vision) [22] while others targeted populations with a variety of developmental disorders who exhibited syndromic elements of ASD, such as fragile X syndrome [23] or Angelman syndrome [24], without studying children with ASD in particular.

This literature review reveals gaps in the current evidence on sensory processing in children with ASD over time and underlines the need for further research on the subject. The main objective of this study was to analyze the evolution of sensory features in children with ASD between the age of 3-4 years (T1) and two years later (T2) when they started school (5-6 years). Our general hypothesis was that overall sensory features of children with ASD would remain stable with age. Our second hypothesis was that the classification of children among the different subcategories of the Sensory Profile would not change between T1 and T2.

## 2. Methods

Three interrelated studies formed the basis of this study. First, between 2007 and 2008, parents of children at the Autism Clinic of the Montreal Children's Hospital were invited to participate in a major pan-Canadian longitudinal study, “Pathways for Better Outcomes” (also known as the Canadian Institutes of Health Research “CIHR-” funded TRAJ project). If they agreed, an ancillary study was conducted only in Montreal to document sensory-motor difficulties and their impact on daily functioning [25]. Additional funding from the FRQSC* (Fonds de Recherche du Québec-Société et Culture)* was obtained for a 2-year follow-up of participants in the sensory-motor study (2010). In the present study, we undertook a secondary analysis of data from both sensory-motor prospective studies (2008–2010). This study was approved by the ethics committee of the* CIUSSS de l'Estrie-CHUS* of the* Université de Sherbrooke* in October 2016.

### 2.1. Participants

Sixty-eight children aged 3 to 4 years diagnosed with ASD who were patients at the Autism Clinic of the Montreal Children's Hospital participated in the sensory-motor study at T1 (2008). Of the original 68, 39 participated in the follow-up two years later (T2). Complete data for the two-time points were available for 34 participants.

The diagnosis was based on the DSM-IV-TR [26] diagnostic criteria, expert clinical judgement, and scores from gold standard diagnostic tools: the Autism Diagnostic Interview, Revised (ADI-R) [27] and the Autism Diagnostic Observation Schedule-Generic (ADOS-G) [28].

### 2.2. Measures

The families' sociodemographic characteristics (child's gender and age, family income, mother's education, marital status, and ethnicity) were collected using a sociodemographic questionnaire designed for this purpose. To characterize the children's cognitive level and language skills and to measure sensory processing over time, the following standardized assessment tools were used:

1. The Merrill-Palmer-Revised Scales of Development (M-P-R) [29] can be used to assess children's development in five domains: cognitive, language, motor, self-reliance, and socioemotional. In this paper, only the standardized score for the cognitive component was used to evaluate cognitive level at T1 (controlled variable). The M-P-R has very good psychometric properties, including internal consistency (*α* = 0.90) and test-retest reliability (intraclass correlation coefficient: ICC = 0.87–0.90) [29].
2. The Preschool Language Scale, Fourth Edition (PLS-4) [30] is an interactive assessment of developmental language skills, including auditory comprehension and expressive communication for children aged 0–7 years [30]. In our study, standard scores were considered for the statistical analyses. Psychometric properties of the test include high interrater reliability (ICC = 0.99) [31].
3. Autism severity scores [32] were calculated using the ADOS severity scores [33]. These calibrated scores range from 1 to 10 and are useful in comparing assessments across modules and over time [33].
4. The Sensory Profile (SP) [34], children's version 3–10 years, is a questionnaire measuring children's response to sensory events that influence their functioning in everyday life. It is completed by parents and includes 125 items. The parent scores the items on a Likert scale (from 1 to 5) depending on the observed frequency of behavior. The questionnaire is divided into subcategories involving sections and quadrants. Sections reflect the different sensory systems (auditory processing, visual processing, etc.) and quadrants refer mainly to the different types of sensory processing (seeking, avoidance, sensitivity, and low registration), which correspond to Dunn's model of sensory processing [12], thus identifying the sensory system(s) that affect daily functioning [35].

More specifically, for each subcategory (sections and quadrants), the SP makes it possible to compare children's results with standardized values in order to classify their performance in three categories: typical performance, probable difference (one standard deviation below the mean), and definite difference (two standard deviations below the mean) [34]. However, the SP does not yield a total score. Finally, the SP has robust psychometric properties for both internal consistency (*α* = 0.89–0.95) [36] and test-retest reliability (ICC = 0.80–0.90) [36].

The Short Sensory Profile (SSP) [18] is a screening tool derived from the SP that has a similar structure to its longer counterpart. Its 38 items are related to the construct of sensory processing and were extracted from the SP. Interestingly, further validation analyses have been done with the SSP, allowing a validated total score to be calculated, which is an asset for quantitative statistical analyses. The SSP allows the calculation of a total score and has acceptable construct validity and its internal consistency is excellent, with *α* = 0.70–0.90 [18].

### 2.3. Procedure

At T1, a lengthy evaluation took place at the Montreal Children's Hospital within the framework of the TRAJ project. An experienced child psychiatrist confirmed the ASD diagnosis, and assessments of cognitive level and language skills were conducted by certified therapists trained in the administration of these tests.

Sensory features were assessed using the caregiver questionnaires. The SP was completed by either parent at the hospital, with the help of two certified occupational therapists, who had more than five years of clinical experience and were also in charge of obtaining signed informed consent from the parents and answering any questions they had.

A nonrandomized convenience sample was used for the families who agreed to participate in the follow-up at T2. After the evaluation was completed at T1, the parents received a report and were invited to participate in a second evaluation of their child's sensory features using the SP two years later (T2). The mean interval between T1 and T2 was 27.7 months (SD = 6.0). In our study, the SSP itself was not administered; instead its 38 items were extracted directly from the responses to the SP. A score was calculated for each subcategory of the SP and a total score was determined for the SSP at both T1 and T2.

### 2.4. Analyses

We analyzed data over time using SPSS 24^©^ software to generate descriptive and inferential statistical analyses. Descriptive analyses for the continuous variables (means and standard deviations) and ordinal variables (frequencies and percentages) were performed on the sociodemographic variables and some of the sensory measurement categories. We verified the normality of the data using the Shapiro-Wilk test and the equality of the variances with Mauchly's sphericity test.

For the inferential analyses, we employed three tests: (1) a repeated measures ANOVA (T1 versus T2) for a single group, with matched measurements, to evaluate the effect of time on the SSP total score and the scores for different SP categories; (2) a repeated measures ANCOVA with the same purpose as the ANOVA, but adding two covariates: cognitive level and autism severity scores (from ADOS-2) [37]; (3) McNemar's tests which were applied to analyze matched categorical data for both time points, as performances on the SSP total score and SP subcategory scores were categorized as either typical for those within the “normal performance” range or atypical for those within the “probable differences” or “definite differences” range. The significance threshold for all tests was set at 0.05. 

## 3. Results

Study population characteristics are presented in Table 1. The sample had a mean age of 45 months at T1 and 72 months at T2; most of the children were boys (82%) with English as their mother language (68%). Most mothers were Caucasian (68%) and had attended university (71%). A large percentage of the mothers (85%) lived with a partner (married, common-law, etc.), and in 77% of the cases, family income was above 40,000 CAD per year (30,000 USD).

Most children had cognitive and language delays as well as a moderately severe level of autism. Seventy-one percent (71%, *n* = 24) presented a significant cognitive delay (standard score < 70), 59% (*n* = 20) had an expressive language delay (standard score < 70), and 71% (*n* = 24) had a receptive language delay. The mean ADOS severity score of the sample was 7.4 (SD = 2.1), which is between a moderately severe and severe level of autism.

Table 2 presents the mean raw scores and results of the repeated measures ANOVAs for the SSP and various SP subcategories at T1 and T2. The effect of time on the SSP total score was not significant (*F*(1,33) = 0.970; *p* = 0.330). The same was true for all SP quadrants and sections. Quadrant 4 (sensory avoiding) (*F*(1,29) = 1.717; *p* = 0.199) was the only mean score to fall within a “typical” range at T2.

In the repeated measures ANCOVAs, there was a nonsignificant effect of time on the SSP score after controlling for the ADOS severity score (*F*(1,28) = 0.087; *p* = 0.771) as well as when controlling for cognitive level (*F*(1,28) = 0.530; *p* = 0.473). The effect of time on the SP subcategories was also not significant (results available upon request).

Our study included 34 pairs of measurements. Our data indicate that the difference in response of matched pairs has a standard deviation of *σ* = 15.34. We detected a true difference in the mean response of matched pairs of −7.596 or 7.596 with a probability of 0.8 (*α* = 0.05). Therefore, the observed stability of scores or absence of a difference between T1 and T2 could be attributed to a lack of power due to the small sample size. However, the magnitude of the difference is so small that it can reasonably be judged to be clinically nonsignificant (smaller than 1/4 SD). However, a larger sample would have allowed for the detection of smaller size effects that could not be detected in our study. Had our study had a larger sample (e.g., *n* = 300), we would have found the clinically nonsignificant differences between T1 and T2 to be statistically significant.

Table 3 presents the findings of the exact McNemar's tests on the classification into two categories of typical-atypical scores for the SSP total score and SP subcategory scores at T1 and T2. Thirty children (*n* = 30, 89%) at T1 and 28 children (82%) at T2 had sensory features (namely, at least one atypical score on the SP or SSP). The exact McNemar's tests determined that there was no statistically significant difference in the proportion of children with at least one atypical score from T1 to T2 (*p* = 0.688). Twenty-two children (*n* = 22; 65%) at T1 and 20 children (59%) at T2 had at least one score in the definite difference category (*p* = 0.754). The percentage of children classified in the atypical category according to the SSP total score increased from 50% (T1) to 56% (T2) but this difference was not statistically significant (*p* = 0.754). The same was true for the SP quadrants and sections. For example, 15 of the 34 participants (44%) had atypical responses for Quadrant 4 (sensory avoiding) at T1 and 13 (38%) at T2 (*p* = 0.774).

We found no statistically significant differences in the SP subcategories (atypical versus typical) when comparing T1 and T2. However, we saw a nonsignificant trend toward improvement over time in 7 (78%) of the 9 SP subcategories while 2 (22%) subcategories deteriorated between T1 and T2.

## 4. Discussion

As discussed in the Introduction, we had hypothesized that overall sensory features of children with ASD would remain stable with age. According to our results, the scores (SP and SSP) remained stable over time. The SSP mean score was in the atypical range at T1 and T2, indicating general sensory features in our study population at both time points. Our results are comparable to other studies which yielded prospective results [16, 17, 19]. Our findings are also in agreement with those in the longitudinal studies by McCormick et al. [17], Ausderau et al. [16], and Green et al. [19], who found that sensory features in children with ASD were stable, and in disagreement with the findings of Ben-Sasson's meta-analysis [21]. Furthermore, since children with typical development (TD) improve their sensory skills with age due to maturation [17, 38], the lack of improvement in sensory skills in children with ASD could mean a relative increase in sensory problems over time in these children.

Despite methodological differences between the longitudinal studies, the conclusion remains the same: sensory features in children with ASD are stable over time. The studies by McCormick et al. [17] and Ausderau et al. [16] also addressed general sensory features even if they used different measuring tools or had more variability in the initial ages than in our study. On the other hand, the study by Green et al. [19] did not measure sensory features in general but sensory overresponsiveness.

Lastly, among the conclusions in Ben-Sasson's study [21] regarding sensory features, they found an increase in the difference between children with ASD and typically developing children up to the age of 9 and a decrease thereafter. This could also be interpreted as an increase in the gap between ASD and TD children over time. Given that the sensory processing skills of typically developing children are expected to improve with age [17, 38], Ben-Sasson's results do not necessarily imply an increase in sensory features in children with ASD. Hence, it is not clear whether this increase was due to a deterioration in sensory features in children with ASD or to an improvement in typical developing children's sensory features. Consequently, our results are not necessarily in conflict with those of Ben-Sasson et al. but could be congruent: sensory features of children with ASD could have remained stable while the increase in the difference could be due to an improvement in sensory skills in children with TD.

We also hypothesized that the classification of children among the SP's different quadrants and sections would not change between T1 and T2. As verified by the ANOVA test, the means of each of the nine subcategories remained stable from T1 to T2, which is a novel finding. Secondly, McNemar tests were run on each SP subcategory, comparing the percentage of children classified as atypical for each subcategory at T1 and T2, further suggesting that the stability of the classification was reinforced.

Our study population was similar to comparable studies [16, 17]: most of the children were Caucasian English-speaking boys from families with a middle-class income and whose mothers had some university education on average. Most children in our study had significant sensory features at an early age, namely, the age of 3-4 years (88%), and these difficulties remained high and stable at least until the age of 6, which suggests that aging does not influence the presence of sensory features. Moreover, the few children with ASD but no sensory features at an early age (12%) did not seem to acquire them two years later, which had not been reported previously. In conclusion, the high frequency of sensory features highlights the need for early interventions addressing these features.

Additionally, the percentage of children classified as having “atypical” scores according to the SSP was stable between T1 (50%) and T2 (56%). Furthermore, if we take into consideration the children who had at least one atypical sensory score between T1 (88.2%) and T2 (82.4%), these percentages were also stable. This was also the case for the “definite difference” category (64.7% at T1 to 58.8% at T2).

Previous studies had pointed to the importance of follow-up periods longer than one year [16, 19]. Therefore, our time of 2 years between measures is an interesting contribution to the scientific literature evaluating the stability of general sensory features. An even longer follow-up period and a larger sample would probably be useful in assessing whether these results continue to be maintained over time. We deliberately designed the study to obtain a fairly high level of homogeneity in age at the beginning of the study and achieved a mean age of 44.7 months (SD = 4.9). Other studies had similar initial homogeneity in age, ranging from 26 to 41 months and from 18 to 33 months, respectively, for McCormick et al. (2016) [17] and Green et al. (2012) [19]. The study by Ausderau et al. (2014) [16] had a wider initial age range, namely, from the age of 2 to 12 years. Changes in the initial T1 age of the study may have important implications on the variation between the two measurements. Younger children aged 2-3 years may have more changes in their sensory information processing skills than older children, say, between 7 and 8 years of age.

We also tested the potential effect of two covariates on the measurement of sensory features over time: the ADOS severity scores and cognitive level (M-P-R). We considered other longitudinal studies [16, 17] that had controlled for different covariates: cognitive level, ASD severity, chronological age, gender, family income, mother's education, and so forth. Based on the literature, we included two previously tested covariates: cognitive level and ASD severity. Like previous studies, we found that the stability of the scores is independent of these two covariates.

Since the results of the present study show that children with ASD display early sensory features that persist, we believe that early detection, diagnosis, and management of ASD and its accompanying sensory features are important. Early treatment is advantageous given younger children's greater neuronal plasticity, which may contribute to improving outcomes [39–41]. Indeed, several studies have provided sufficient evidence in support of interventions targeting ASD [42], including behavioral interventions [43], the cognitive behavioral intervention package, language training, modeling, parent training [44], and the peer training package [42]. To date, there is no single guideline on how best to support children with sensory features. There is a growing amount of promising but still contradictory evidence regarding the efficacy of treatments to improve sensory features in children with ASD [6, 35, 45–48], and no specific strategy can be recommended as yet. However, parents often seek out OT services to better understand their child's sensory processing skills and to be able to modify tasks or structure the environment differently to facilitate the sensory processing of their child. Therefore, if children with ASD have true stable sensory processing difficulties despite the very marginal early interventions received and not specific to this area of their development, we believe it is still appropriate to recommend early intervention targeting specifically strategies to compensate by modifying the environment, tasks, or expectations (e.g., avoiding noisy places for a child with auditory hypersensitivity) to improve the quality of life of the child and the family.

## 5. Conclusions

Children with ASD have sensory features starting at an early age, namely, before 3-4 years, and continue to have these difficulties two years later when they start school (at the age of 5-6 years). We showed that the SP quadrants and sections were also remarkably stable over time, with no independent effect of time once we controlled for cognitive level and autism severity. Furthermore, the expected improvement in sensory skills in children with TD is not seen in children with ASD, which could mean an increase in their sensory features. Further research should seek to address our study's limitations by increasing follow-up time, adding a control group, controlling for the treatment received, and increasing the size of the sample to detect smaller effects size.

In conclusion, sensory features in children with ASD begin early in life and persist. Knowing that sensory features have a negative impact on daily life skills [25], anxiety [19], and social integration of children with ASD [46], we believe that efforts should be made to ensure early recognition and management of these sensory features to improve their functional and psychosocial outcomes.

## Figures and Tables

**Table 1:** General characteristics of participants.

	*n*	%	Mean (SD)
Gender			
Boys	28	82.4	
Girls	6	17.6	
Chronological age			
T1 (months)	34		44.7 (4.9)
T2 (months)	34		72.4 (5.4)
ADOS comparison score (CSS^a^)	34		7.4 (2.1)
Cognitive functioning			
M-P-R SS^b^	32		60.1 (25.8)
Significant cognitive delay	24	70.6	
Language skills			
PLS-4 auditory SS	33		63.4 (21.9)
Significant auditory delay	20	58.8	
PLS-4 expressive SS	33		70.6 (19.9)
Significant expressive delay	24	70.6	
Mother's education			
University	24	70.6	
No university	10	29.4	
Mother's ethnicity			
Caucasian	23	67.6	
Non-Caucasian	11	32.4	
Mother's marital status			
Single	5	14.7	
Married/common-law	29	85.3	
Family income			
>40,000 CAD	26	76.5	
<40,000 CAD	8	23.5	
Mother tongue			
English	23	67.6	
French	4	11.8	
Other	7	20.6	

ASD rated from 1 to 10, with 10 being the most severe; ^a^CSS is calibrated severity scores; ^b^SS is standard score; M-P-R: Merrill-Palmer-Revised Scales of Development; PLS-4: Preschool Language Scale, Fourth Edition.

**Table 2:** Repeated measures ANOVA results comparing T1 and T2.

	Mean raw score T1 (SD)	Mean raw score T2 (SD)	*F* ^a^	*p* value^b^
SSP total score	**153.9 (12.6)**	**151.4 (16.6)**	0.970	0.330
SP quadrants				
(Q1) Low registration	**63.3 (6.5)**	**63.9 (7.8)**	0.180	0.674
(Q2) Sensory seeking	**98.2 (13.7)**	**99.2 (19.3)**	0.180	0.674
(Q3) Sensory sensitivity	**78.6 (9.1)**	**78.8 (10.8)**	0.006	0.940
(Q4) Sensory avoiding	**112.0 (14.9)**	115.6 (11.9)	1.717	0.199
SP sections				
(A) Auditory processing	**28.5 (6.2)**	**29.8 (5.1)**	2.463	0.126
(B) Visual processing	37.2 (5.4)	38.4 (5.1)	2.023	0.164
(C) Vestibular processing	**47.1 (3.7)**	**47.5 (5.9)**	0.188	0.667
(D) Touch processing	73.1 (9.1)	74.3 (10.6)	0.681	0.415
(E) Oral sensory processing	46.9 (8.7)	**44.9 (10.6)**	1.510	0.228

^a^Repeated measures ANOVAs (tests of within-subjects effects); ^b^*p* < 0.05. *Note*. Means in the atypical range are shown in bold.

**Table 3:** McNemar sensory results at T1 and T2.

Variable	ASD atypical responses T1		ASD atypical responses T2		*χ* ^2^ McNemar *p* value^a,b^
	*n*	%	*n*	%	
SSP total score	17	50.0	19	55.9	0.754
At least one “atypical category”	30	88.8	28	82.4	0.688
At least one “definite difference”	22	64.7	20	58.8	0.754
SP quadrants					
(Q1) Low registration	14	41.2	13	38.2	1.000
(Q2) Sensory seeking	20	58.8	18	52.9	0.727
(Q3) Sensory sensitivity	16	47.1	17	50.0	1.000
(Q4) Sensory avoiding	15	44.1	13	38.2	0.774
SP sections					
(A) Auditory processing	18	52.9	16	47.1	0.774
(B) Visual processing	4	11.8	3	8.8	1.000
(C) Vestibular processing	16	47.1	15	44.1	1.000
(D) Touch processing	14	41.2	13	38.2	1.000
(E) Oral sensory processing	12	35.3	16	47.1	0.344

^a^2-tailed binomial distribution used; ^b^*p* < 0.05.
